# Ring-Expansion Reaction of Oximes with Aluminum Reductants

**DOI:** 10.3390/molecules17067348

**Published:** 2012-06-14

**Authors:** Hidetsura Cho, Yusuke Iwama, Nakako Mitsuhashi, Kenji Sugimoto, Kentaro Okano, Hidetoshi Tokuyama

**Affiliations:** 1 Graduate School of Science, Tohoku University, 6-3 Aoba, Aramaki, Aoba-ku, Sendai 980-8578, Japan; 2 Graduate School of Pharmaceutical Sciences, Tohoku University, 6-3 Aoba, Aramaki, Aoba-ku, Sendai 980-8578, Japan

**Keywords:** aluminum reductant, dichloroaluminum hydride (AlHCl_2_), ring-expansion of oxime, rearrangement of oxime, cyclopentyl methyl ether (CPME)

## Abstract

The ring-expansion reactions of heterocyclic ketoximes and carbocyclic ketoximes with several reductants such as AlHCl_2_, AlH_3_ (alane), LiAlH_4_, LiAlH(O*^t^*Bu)_3_, and (MeOCH_2_CH_2_O)_2_AlH_2_Na (Red-Al) were examined. Among reductants, AlHCl_2_ (LiAlH_4_:AlCl_3_ = 1:3) in cyclopentyl methyl ether (CPME) has been found to be a suitable reagent for the reaction, and the rearranged cyclic secondary amines were obtained in good to excellent yields.

## 1. Introduction

The development of novel synthetic method of constructing basic heterocyclic skeletons is an important research topic from the viewpoint of both synthetic chemistry and medicinal chemistry. Specifically, the fundamental skeletons containing a nitrogen functionality attached to an aromatic ring are of great importance because they are often used as the core structures of medicines or clinical candidates. In this research area, we have recently reported the synthesis of five- to eight-membered bicyclic or tricyclic fused heterocycles containing nitrogen attached to an aromatic ring by the reductive ring expansion reaction of cyclic ketoximes or hydroxylamines using diisobutylaluminum hydride [DIBALH: (*^i^*Bu)_2_AlH] [1,2,3,4,5,6]. We also carried out mechanistic studies to prove the intermediacy of the corresponding hydroxylamines and to obtain mechanistic information about the ring expansion on the basis of DFT calculations [3].

However, we have not yet performed systematic examinations of suitable reductants and solvents for the reductive ring expansion reaction. A similar reaction using borane was in fact reported by Ortiz-Marciales *et al.* The reductive ring expansion of *O*-silylated oximes proceeded using borane in the presence of boron trifluoride [7]. In this report, we disclose our recent results on the reductive ring-expansion reactions of oximes with a variety of aluminum reductants.

## 2. Results and Discussion

We selected five reductants, *i.e.*, lithium aluminum hydride (LiAlH_4_) [8,9], aluminum hydride (AlH_3_; alane) [9,10,11], sodium bis(2-methoxyethoxy)aluminum hydride (Red-Al; Vitride) [12], dichloroaluminum hydride (AlHCl_2_) [9,10,11,13], lithium tri-*tert*-butoxyaluminum hydride [LiAlH(O*^t^*Bu)_3_], and compared their reactivities using the oxime **1a** as the test substrate (Table 1).

**Table 1:** Rearrangement of oxime with various reductants. 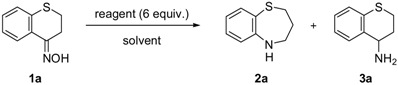

Entry	Reagent	Solvent	Temp.	Time	2a (%)	3a (%)
1	LiAlH_4_	Et_2_O	0 °C to rt	5 h	29	45
2	(MeOCH_2_CH_2_O)_2_AlH_2_Na	toluene	0 to 50 °C	5 h	31	18
3	LiAlH(O*^t^*Bu)_3_	Et_2_O	0 °C to reflux	24 h	0	0
4	AlH_3_	Et_2_O	0 °C to rt	2 h	46	47
5	AlHCl_2_	Et_2_O	0 °C to rt	2 h	72	6
6	AlHCl_2_	CPME	0 °C to rt	2 h	76	0

**Scheme 1:**
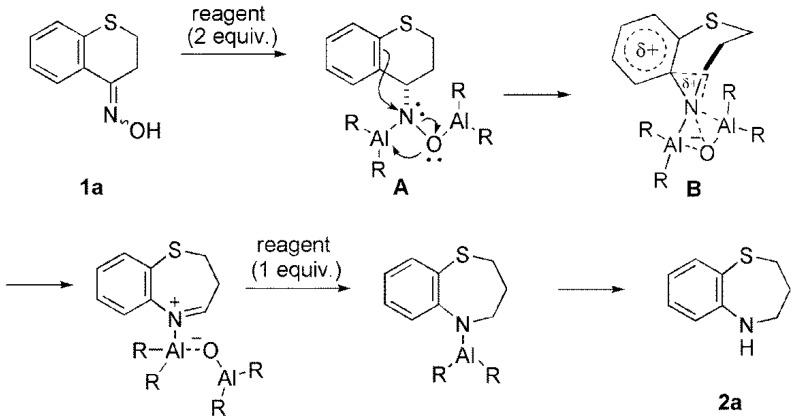
Proposed mechanisms of reductive ring expansion reaction of ketoximes with the aluminum reagent.

When **1a** was treated with six mol equiv. of LiAlH_4_, the desired ring expansion product, 2,3,4,5-tetrahydrobenzo[*b*][1,4],thiazepine (**2a**) was obtained in only 29% yield and was associated with substantial amounts of the primary amine **3a**[14], which should be generated by the C=N and N–O reduction of the oxime (Entry 1). Reaction with Red-Al gave similar results providing a mixture of **2a** (31%) and **3a** (18%) (Entry 2). LiAlH(O*^t^*Bu)_3_, on the other hand, was considerably less reactive and produced no product (Entry 3). Next, we examined AlH_3_ and AlHCl_2_, which possess Lewis acidic character. When **1a** was treated with six mol equiv. of AlH_3_ in Et_2_O, a result parallel to those of LiAlH_4 _andRed-Al was obtained. Thus, a mixture of **2a** and **3a** in 46% and in 47% yield, respectively, was isolated (Entry 4). Interestingly, however, the treatment of the ketoxime **1a** with six mol equiv. of AlHCl_2_, which was prepared as a suspension in Et_2_O, afforded **2a** in 72% yield associated with only a small amount of the primary amine **3a** (6%) (Entry 5). The smooth ring expansion after 1,2-reduction may be attributed to the Lewis acidity of AlHCl_2_ *etc.*, which should coordinate with the oxygen of the hydroxylamine **A** to promote a rearrangement process via intermediate **B** (Scheme 1) [3]. Having found that AlHCl_2_ is a suitable reductant to promote the ring expansion reaction, we then investigated this generality along with solvent effects. As to reaction solvents, several solvents such as Et_2_O, *^i^*Pr_2_O, THF, cyclopentyl methyl ether (CPME) [15,16] and mixed solvents were examined. Among them, the use of CPME was found to suppress the formation of undesired **3a** to provide **2a** in 76% yield (Entry 6). CPME is an alternative to conventional ethereal solvents, such as THF and diethyl ether, due to a higher solubility for substrates, the superior handling, and safety for a large-scale production [15].

The generality of CPME was examined using a variety of cyclic ketoximes (Table 2). Although the reaction of **1b** in CPME provided 2,3,4,5-tetrahydrobenzo[*b*][1,4]oxazepine (**2b**) in slightly lower yield than in Et_2_O (Entry 2), reactions using **1a**, **1c**, and **1d** in CPME afforded 2,3,4,5-tetrahydro-benzo[*b*][1,4]thiazepine (**2a**), 2,3,4,5-tetrahydro-1*H*-benz[*b*]azepine (**2c**), and 5,6,7,8-tetrahydro-4*H*-thieno[3,2-*b*]azepine (**2d**) in much better yields (Entries 1, 3, and 4), respectively. In addition, the reactions of aryl oximes **1e** and **1f** furnished the desired tetrahydrobenzoazepines **2e** and **2f** in good to excellent yields (Entries 5 and 6). Subsequently, we applied the reaction to five- or seven-membered oximes. While the reaction of **1g** in Et_2_O gave 1,2,3,4-tetrahydroquinoline (**2g**) in moderate yield because of the recovered starting material, the reaction in CPME provided **2g** in better yields than in Et_2_O (Entry 7). The treatment of **1h** with AlHCl_2_ in CPME also gave 1,2,3,4,5,6-hexahydrobenz[*b*]azocine (**2h**) in good yield (Entry 8)*.*

**Table 2:** Rearrangement of oxime with dichloroaluminum hydride. 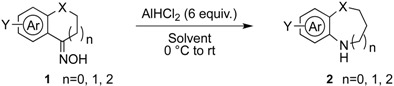

Entry	Oxime 1	Solvent	Rearranged Product 2	Yield of 2
1	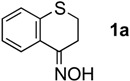	Et_2_O	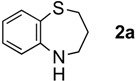	72%
		CPME		76%
2	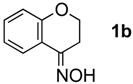	Et_2_O	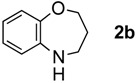	87%
		CPME		83%
3	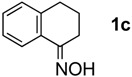	Et_2_O	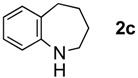	54%
		CPME		78%
4	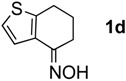	Et_2_O	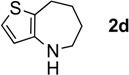	68%
		CPME		88%
5	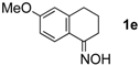	CPME	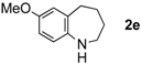	84%
6	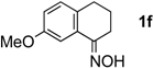	CPME	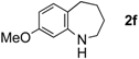	78%
7	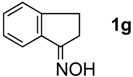	Et_2_O	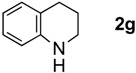	45%
		CPME		69%
8	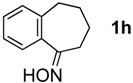	CPME	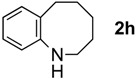	69%

## 3. Experimental

### 3.1. General

All the melting points were determined with a Yanaco micro melting point apparatus and are uncorrected. IR spectra were measured with a Shimadzu FTIR-8300 spectrometer. NMR spectra (at 400 MHz for ^1^H and 100 MHz for ^13^C) were recorded on a JEOL JNM-Al 400 spectrometer with tetramethylsilane (0 ppm) or chloroform (7.24 ppm) as the internal standard. Mass spectra were recorded on JMS-DX303, JMS-700, or JMS-T100GC spectrometers. Elemental analyses were performed with a Yanaco CHN CORDER MT-6. Column chromatography was performed on silica gel 60N (Kanto, 63–210 μm), and flash column chromatography was performed on silica gel 60N (Kanto, 40–60 μm) using the indicated solvents. Reactions and chromatography fractions were monitored by using precoated silica gel 60 F_254_ plates (Merck).

### 3.2. General Preparation of AlHCl2 and AlH3 in Accordance with the Procedure Reported by Ashby et al. *[10,11]*

Four mol equiv. of AlHCl_2_ (containing one mol equiv. of LiCl) was prepared in Et_2_O or CPME at 0 °C from one mol equiv. of LiAlH_4_, and three mol equiv. of AlCl_3_. Four mol equiv. of AlH_3_ (containing three mol equiv. of LiCl) was prepared in Et_2_O from three mol equiv. of LiAlH_4_ and one mol equiv. of AlCl_3_.

#### 3.2.1. Synthesis of 2,3,4,5-tetrahydrobenzo[b][1,4]thiazepine (**2a**) and 3,4-dihydro-2H-thiochromen-4-ylamine (**3a**)

Reaction of **1a** with 6.0 mol equiv. of AlHCl_2_ in CPME (Table 2, Entry 1). A flame-dried 30-mL two-necked round-bottomed flask equipped with a magnetic stirring bar was charged with LiAlH_4_ (15.0 mg, 395 µmol). The LiAlH_4_ in the flask was stirred at 0 °C. A dry CPME (1.5 mL) solution of AlCl_3_ (159 mg, 1,190 μmol) from a flame-dried 10-mL two-necked round-bottomed flask was slowly added to the reaction mixture over a period of 5 min by cannulation. The reaction mixture was stirred at 0 °C for 2 h. Thiochroman-4-one oxime (**1a**, 47.3 mg, 264 µmol) in dry CPME (2.5 mL) from a flame-dried 10-mL two-necked round-bottomed flask was added slowly to the reaction mixture over a period of 5 min by cannulation. After stirring for 10 min at 0 °C, the reaction mixture was warmed to room temperature, stirred for another 2 h, cooled to 0 °C, and then treated carefully with wet Et_2_O (2 mL) and water (2 mL). The mixture was made basic with 1 M aqueous potassium hydroxide (5 mL) and extracted with ethyl acetate. The combined organic extracts were dried over anhydrous sodium sulfate and filtered. The filtrate was concentrated under reduced pressure. The residue was purified twice by preparative TLC (hexane/EtOAc = 5:1) to afford pure 2,3,4,5-tetrahydrobenzo[*b*][1,4]thiazepine (**2a**) (33.2 mg, 201 µmol, 76%) as a yellow oil. To a solution of **2a** in Et_2_O was added hydrochloric acid in Et_2_O (1 M) at room temperature. After stirring, Et_2_O was removed under reduced pressure. The residue was purified by recrystallization to give the hydrochloric acid salt of **2a** as colorless crystals.

*2*,*3*,*4*,*5-Tetrahydrobenzo[*b][1,4] *thiazepine* (**2a**) *hydrochloride*. M.p.: 138–142 °C (from EtOH), m.p. 142–144 °C (from *i*-PrOH). ^1^H-NMR (CD_3_OD): δ 7.74 (dd, 1H, *J* = 1.6 and 7.6 Hz), 7.56 (dd, 1H, *J* = 1.6 and 7.6 Hz), 7.51 (ddd, 1H, *J* = 1.6, 7.6 and 7.6 Hz), 7.46 (ddd, 1H, *J* = 1.6, 7.6 and 7.6 Hz), 3.51 (t, 2H, *J* = 5.6 Hz), 2.95 (t, 2H, *J* = 5.6 Hz), 2.40 (tt, 2H, *J* = 5.6 and 5.6 Hz). ^13^C-NMR (CD_3_OD): δ 140.9, 136.0, 132.6, 131.3, 131.0, 124.7, 50.9, 32.7, 29.9. IR (KBr, cm^−1^): 2914, 2687, 1558, 1456, 764. Elemental analysis: calcd. (%) for C_9_H_12_ClNS: C 53.59, H 6.00, N 6.94. Found: C 53.47, H 5.85, N 6.89.

Orlova *et al.* carried out the reaction of **1a** with the reagent LiAlH_4_-AlCl_3_ (1:4, 4 equiv. to **1a**) and described that **2a** was obtained in 80.5% yield. The melting point (m.p. 202–204 °C from *i*-PrOH) of the HCl salts reported is different from our HCl salts (m.p. 142–144 °C from *i*-PrOH) [13]. 

Reaction of **1a** with 6.1 mol equiv. of LiAlH_4_ (Table 1, Entry 1). A flame-dried 10-mL two-necked round-bottomed flask equipped with a magnetic stirring bar was charged with LiAlH_4_ (23.2 mg, 610 μmol). The LiAlH_4_ in the flask was stirred at 0 °C. To the stirred LiAlH_4_ was added dry Et_2_O (1.0 mL). To the suspension was added **1a** (18.2 mg, 100 μmol). After stirring for 0.5 h at 0 °C, the reaction mixture was warmed to room temperature, stirred for another 6 h, cooled to 0 °C, and then treated carefully with wet Et_2_O (1 mL) and water (1 mL). The mixture was made basic with 2 M aqueous NaOH (2 mL) and extracted with Et_2_O. The combined organic extracts were washed with brine, dried over anhydrous sodium sulfate, and filtered. The filtrate was concentrated under reduced pressure. The residue was purified by preparative TLC (hexane/Et_2_O = 3:1) to afford **2a** (4.8 mg, 29 µmol, 29%) and **3a** (7.4 mg, 45 µmol, 45%).

*3*, *4-Dihydro-2H-thiochromen-4-ylamine ***(3a)**[14]; ^1^H-NMR (CDCl_3_): δ 7.32–7.23 (m, 1H), 7.16–7.00 (m, 3H), 4.05 (brs, 1H), 3.31–3.19 (m, 1H), 2.98–2.87 (m, 1H), 2.17–2.05 (m, 2H), 1.62 (br s, 2H). ^13^C-NMR (CDCl_3_): δ 137.4, 132.3, 129.2, 127.4, 126.7, 124.2, 48.4, 31.0, 22.1. IR (neat, cm^-1^): 2920, 2849, 1583, 1566, 1472, 1435, 1286, 1074, 1042, 887, 754, 731. HRMS-EI calcd. for C_9_H_11_NS (M^+^) 165.0612. Found: 165.0608. 

Reaction of **1a** with 6.0 mol equiv. of Red-Al **(Table 1**, Entry 2). A two-necked 10-mL round-bottomed flask equipped with a magnetic stirring bar was charged with **1a** (18.0 mg, 100 μmol) and dry toluene (1 mL). The solution was cooled to 0 °C. To the solution was added Red-Al (76 μL, ≥65 wt% in toluene, 600 μmol) at 0 °C, and the resulting mixture was stirred at room temperature for 0.5 h. The reaction mixture was heated at 50 °C for 6 h, cooled to 0 °C, and then treated carefully with wet Et_2_O (1 mL) and water (1 mL). The mixture was made basic with 2 M aqueous NaOH (2 mL) and extracted with Et_2_O. The combined organic extracts were washed with brine, dried over anhydrous sodium sulfate, and filtered. The filtrate was concentrated under reduced pressure. The residue was purified by preparative TLC (hexane/Et_2_O = 3:1) to afford **2a** (5.1 mg, 31 µmol, 31%) and **3a** (2.9 mg, 18 µmol, 18%). Orlova and Kucherova reported the reaction of **1a** with Red-Al, but they simply noted the reaction in only 12 lines and no details were given [12].

Reaction of **1a** with 5.9 mol equiv. of AlH_3_ (Table 1, Entry 4). A flame-dried 10-mL two-necked round-bottomed flask equipped with a magnetic stirring bar was charged with LiAlH_4_ (16.8 mg, 443 μmol). The LiAlH_4_ in the flask was stirred at 0 °C. To the stirred LiAlH_4_ was added dry Et_2_O (1.0 mL) and AlCl_3_ (23.2 mg, 170 μmol). The reaction mixture was stirred at 0 °C for 1 h. To the suspension was added **1a** (18.2 mg, 100 μmol). After stirring for 0.5 h at 0 °C, the reaction mixture was warmed to room temperature, stirred for another 2 h, cooled to 0 °C, and then treated carefully with wet Et_2_O (1 mL) and water (1 mL). The mixture was made basic with 2 M aqueous NaOH (2 mL) and extracted with Et_2_O. The combined organic extracts were washed with brine, dried over anhydrous sodium sulfate, and filtered. The filtrate was concentrated under reduced pressure. The residue was purified by preparative TLC (hexane/Et_2_O = 3:1) to afford **2a** (7.6 mg, 46 µmol, 46%) and **3a** (7.8 mg, 47 µmol, 47%). 

#### 3.2.2. Synthesis of 5,6,7,8-tetrahydro-4H-thieno[3,2-b]azepine (**2d**)

Reaction of **1d** with 6.0 mol equiv. of AlHCl_2_ in CPME (Table 2, Entry 4). To a flame-dried 100-mL two-necked round-bottomed flask equipped with a magnetic stirring bar were successively added LiAlH_4_ (65.1 mg, 1.72 mmol), anhydrous CPME (10 mL), and AlCl_3_ (682 mg, 5.11 mmol) at 0 °C. Stirring was continued at 0 °C for 1 h. 6,7-Dihydro-4-benzo[*b*]thiophenone oxime (**1d**, 167 mg, 1.00 mmol) was added in a small portion. After stirring for 0.5 h at 0 °C, the reaction mixture was warmed to room temperature, stirred for another 2.5 h, cooled to 0 °C, and then treated carefully with wet Et_2_O (10 mL) and 2 M aqueous NaOH (20 mL). The mixture was extracted with Et_2_O and the combined organic extract was washed with brine, dried over anhydrous sodium sulfate, and filtered. The filtrate was concentrated under reduced pressure to give the residue, which was purified by silica gel column chromatography (hexanes/EtOAc = 3:1) to afford 5,6,7,8-tetrahydro-4*H*-thieno[3,2-*b*]azepine (**2d**, 135 mg, 0.881 mmol, 88%) as a yellow oil [1].

## 4. Conclusions

The examination of the reductive ring-expansion reaction of cyclic ketoximes using a variety of aluminum reductants, *i.e.*, LiAlH_4_, LiAlH(O*^t^*Bu)_3_, Red-Al, AlHCl_2_, and AlH_3_, revealed that dichloroaluminum hydride (AlHCl_2_) (LiAlH_4_/AlCl_3_ = 1:3) is a suitable reagent for promoting the reaction and affords ring expansion products in good to excellent yields. In addition, it was clarified that CPME could be effective solvent than Et_2_O for the rearrangement of cyclic ketoximes with AlHCl_2_. The finding may lead to further synthetic application of variously substituted heterocyclic compounds and complicated medicine candidates containing a nitrogen functionality attached to an aromatic ring.
